# VETERAN AND FAMILY PERSPECTIVES OF THE VA NURSING HOME TO HOME PROGRAM

**DOI:** 10.1093/geroni/igae098.4380

**Published:** 2024-12-31

**Authors:** Leah Haverhals, Chelsea Manheim, Rachel Fix

**Affiliations:** Rocky Mountain Regional VA Medical Center, Aurora, Colorado, United States; Rocky Mountain Regional VA Medical Center, Aurora, Colorado, United States; Rocky Mountain Regional VA Medical Center, Aurora, Colorado, United States

## Abstract

Veterans meeting United States (US) Veterans Health Administration (VA) service-connected disability requirements are eligible for VA provided nursing home (NH) care, resulting in high utilization. In 2023, the VA launched the Nursing Home to Home (NHTH) pilot program at three VA Medical Centers. NHTH’s objective is to identify Veterans residing in VA-contracted NHs interested in returning to live in the community. Through assessments by an interdisciplinary team, care coordination, and follow-up services, efforts are initiated to support appropriate, safe, and successful discharges. From March-July 2024, we conducted N=11 interviews with 13 Veterans and family members to describe their experiences with NHTH. Veterans felt more comfortable at home, had adequate caregiving and home health support, and said their healthcare needs were met. Several shared they were confident in bathing and dressing independently and expressed increased self-determination in improving their mobility. Families and Veterans noted the time from program referral to NH discharge ranged from three to six months, which they felt was appropriate. Families shared the NHTH team was available to answer questions, provide education, and offer help navigating the VA, including identifying services Veterans may be eligible for that families had not been aware of previously. One family member shared the NH had not provided adequate care to the Veteran and was glad the Veteran returned home, and many Veterans noted they would recommend the program to other Veterans. After one year, feedback shared about NHTH has been largely positive, highlighting several benefits and showing potential for expansion.

